# Use of starch‐based fat replacers in foods as a strategy to reduce dietary intake of fat and risk of metabolic diseases

**DOI:** 10.1002/fsn3.1303

**Published:** 2019-12-03

**Authors:** Yuwei Chen, Yongbo She, Ruisan Zhang, Jieying Wang, Xiaohua Zhang, Xingchun Gou

**Affiliations:** ^1^ Shaanxi Key Laboratory of Brain Disorders Department of Public Health Xi’an Medical University Xi’an China; ^2^ Metabolic and Cardiovascular Diseases Laboratory Li Ka Shing Centre for Health Research Innovation University of Alberta Edmonton Alberta Canada; ^3^ Shaanxi Key Laboratory of Brain Disorders & Institute of Basic Medical Sciences & Institute of Basic and Translational Medicine Xi’an Medical University Xi’an China

**Keywords:** cardiovascular disease, dietary fat, modified starch, starch‐based fat replacers

## Abstract

Cardiovascular disease (CVD) has emerged as one of the leading causes of death worldwide. Elevated blood cholesterol and low‐density lipoprotein levels are crucial risk factors that contribute to the development of CVD and other metabolic diseases. Dietary fat is believed to be the key factor in modulating circulating cholesterol levels. Thus, reducing dietary intake of fat appears to be an effective strategy to reduce the risk of heart disease. Also, excessive intake of fat and high‐calorie foods is also related to the development of obesity, which contributes to the development of CVD. Therefore, the consumption of low‐fat low‐calorie foods is part of a healthier dietary pattern. However, simply removing fat from foods may lead to compromised overall quality and reduced acceptance of the food products. Thus, fat replacers have emerged as ideal alternatives to dietary fat, which can not only reduce the overall fat and calorie content of the foods but also mimic the physiochemical properties of dietary fat. Starch‐based fat replacers are one kind of fat mimetic that can be produced either chemically as modified starch or enzymatically as maltodextrins. Both modified starch and maltodextrins have been demonstrated to have a promising ability to improve the overall quality of reduced‐fat foods. Modified starch granules act directly as fat globules in modulating the structure and sensory characteristics of the foods, whereas maltodextrins can form thermoreversible gels. Both modified starch granules and maltodextrins can create a fat‐like mouthfeel and therefore are potential fat replacers. This review article aims to discuss the following topics: (a) the effect of carbohydrates and fat on human cardiovascular health and other disease risks, (b) the functionality of starch‐based fat replacers in foods, (c) the applications of starch‐based fat replacers in various foods, and (d) the current and future market value of starch‐based fat replacers.

## Graphical abstract

This review article discusses the following objectives: (a) effect of carbohydrate and fat on human cardiovascular health and other disease risks, (b) functionality of starch‐based fat replacers in foods, (c) applications of starch‐based fat replacers in various foods, and (d) current and future market value of starch‐based fat replacers.

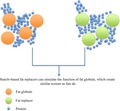

## 1 INTRODUCTION

Cardiovascular health is a concern of many healthcare organizations and scientists worldwide. According to Health Canada, approximately 2.4 million Canadians aged 20 years and older have heart disease (Government of Canada, 2017). Although the pathogenesis of cardiovascular disease (CVD) is complicated and involves several factors, dietary intervention appears to be one of the most popular and easiest strategies to prevent CVD. Reducing circulating levels of total cholesterol (TC) and low‐density lipoprotein cholesterol (LDL‐C) has been considered the primary strategy for controlling the prevalence of CVD in humans. Thus, several dietary guidelines and healthcare organizations have made recommendations such as limiting saturated fatty acid (SFA) intake (<10%/d) and substituting SFAs with polyunsaturated fatty acids (PUFAs) or monounsaturated fatty acids (MUFAs) to lower circulating TC and LDL‐C levels (American Heart Association, 2017).

The quality of dietary fatty acids can differentially modulate circulating cholesterol levels and the risk of CVD and other metabolic diseases. For instance, trans fats were widely used in food manufacturing partially due to their unique physiochemical properties, such as their solid‐like appearance, desirable flavor, and texture (American Heart Association, 2017). Trans fats were widely used in multiple food products, such as baked goods, potato chips, and deep‐fried foods (Dietitians of Canada, 2018). However, trans fats are known to significantly increase the risk of heart disease by increasing circulating TC/LDL‐C levels in humans (Hammad, Pu, & Jones, 2016). Therefore, several authorities, including the FDA and Health Canada, have banned the use of trans fat in all manufactured food products. However, other types of dietary fats can also adversely affect human health. For instance, long‐chain saturated fatty acids (SFAs), myristic acid (C14:0), and palmitic acid (C16:0) are believed to increase circulating TC/LDL‐C levels, increasing the risk of heart disease (Ohlsson, Burling, & Nilsson, 2010; Siri‐Tarino, Sun, Hu, & Krauss, 2010). However, these two long‐chain SFAs are found in large amounts in dairy products. On the other hand, reducing overall calorie intake has always been considered as a strategy to reduce the risk of obesity. Fat contains 9 cal/g energy, which is significantly higher than the calorie content of other macronutrients such as carbohydrates and protein, which only contain 4 cal/g.

Thus, reducing the overall intake of dietary fat and gradually replacing it with other ingredients appear to be a feasible approach to control the prevalence of heart disease, obesity, and other metabolic diseases. However, simply removing fats from food may adversely affect the overall quality of products as fat contributes to the physiochemical properties, such as viscosity, texture, appearance, and flavor, of final food products (Peng & Yao, 2017). Thus, using fat replacers in foods has emerged as a promising tool to reduce fat content in the food industry. Fat replacers are classified as fat substitutes and fat mimetics depending on their chemical conformation and functionalities (Peng & Yao, 2017). The former type of fat replacers aims to replace fat on a “one‐to‐one” basis and has similar chemical conformations as fats, whereas the latter type of fat replacers does not. Briefly, lipid‐based fat replacers are normally considered fat substitutes, whereas carbohydrate‐ and protein‐based fat replacers are considered fat mimetics (Peng & Yao, 2017).

Carbohydrates, a common macronutrient in the human diet that contains less energy than fat, are capable of serving as fat replacers due to their unique physiochemical properties. Carbohydrate‐based fat replacers have been extensively studied and developed for decades, and therefore, various types of commercial carbohydrate‐based fat replacers are currently available on the market. Starch‐based fat replacers are one type of fat replacer; fat is replaced either with native starch or with modified starch. The objectives of the present review are as follows: (a) to compare the effects of carbohydrates and fat on human cardiovascular health and other disease risks, (b) to discuss the functionality of starch‐based fat replacers in foods, (c) to discuss the applications of starch‐based fat replacers in various foods, and (d) to briefly review the current and future market value of starch‐based fat replacers.

## 2 EFFECTS OF DIETARY FAT VERSUS THOSE OF CARBOHYDRATES ON HUMAN CARDIOMETABOLIC HEALTH

The link between dietary fatty acids, circulating cholesterol levels, and heart disease has been extensively studied in recent decades. Modulating dietary fat intake appears to be one of the most effective approaches to control the prevalence of heart disease. A typical type of dietary fat, trans fat, was found to significantly increase TC/LDL‐C levels, contributing to the pathogenesis of atherosclerosis (Hammad et al., 2016). Trans fat has played a crucial role in the food industry for a very long time. However, the FDA announced the ban against hydrogenated fat in food products, which was effective as of June 2018 (Food & Drug Agency, 2018). Similarly, Health Canada banned the use of trans fat in food effective September 2018 (Health Canada, 2018). Nevertheless, there are considerable amounts of other fats present in the daily human diet. For instance, dairy products such as yogurt, cheese, and butter play crucial roles in the North American diet. They are believed to provide probiotics, minerals, vitamins, and proteins to humans. However, a significant amount of dietary fat is also naturally present in all dairy products. The long‐chain SFA palmitic acid is the predominant saturated fat in dairy products, accounting for up to 35% of its total fat content (Hammad et al., 2016). Studies have shown that dietary palmitic acid contributes to elevating circulating TC/LDL‐C levels, increasing the risk of heart disease (Ohlsson et al., 2010; Siri‐Tarino et al., 2010). Similarly, in a clinical study, daily intake of 3 servings of dairy products (1% fat milk, 1.5% fat yogurt, and 34% fat cheese) significantly increased TC/LDL‐C levels compared to the effects of a low‐fat control diet (Hammad et al., 2016). Thus, limiting the intake of SFAs and overall fat content has emerged as one of the strategies to prevent heart disease and other metabolic diseases. The American Heart Association recommends that dietary intake of saturated fat be limited to <10% and gradually replaced by other types of macronutrients (American Heart Association, 2017).

When comparing carbohydrates to dietary fat, it appears that carbohydrates do not increase the risk of heart disease or other metabolic diseases and do not adversely affect blood lipid levels. For instance, the randomized full‐feeding crossover clinical trial with 92 women and men conducted between Laval University and University of Manitoba aimed to investigate the effects of consuming dairy saturated fat on cardiometabolic risk factors compared to the effects of carbohydrates and unsaturated fats (Brassard et al., 2017). A total of 92 subjects were randomized into 5 isocaloric dietary treatment groups: (a) a diet rich in SFAs from cheese, (b) a diet rich in SFAs from butter, (c) a diet rich in MUFAs from olive oil, (d) a diet rich in PUFAs from sunflower oil, and (e) a high‐carbohydrate low‐fat diet. Each of the treatments lasted for 4 weeks, followed by a 4‐week washout period (Babu, Parimalavalli, & Mohan, 2018). Findings from the study showed that serum TC, LDL‐C, and high‐density lipoprotein cholesterol (HDL‐C) levels were significantly reduced in 92 subjects when 6% energy from dairy fat was isocalorically replaced by carbohydrates (Brassard et al., 2017). Similarly, a randomized single‐blinded placebo‐controlled trial with 75 women and men investigated the effect of consuming high‐fat low‐carbohydrate and low‐fat high‐carbohydrate diets on body weight, body composition, and insulin levels (Kahleova, Dort, Holubkov, & Barnard, 2018). Participants were either required to follow their habitual diet or follow the high‐carbohydrate low‐fat diet designed by a registered dietitian for 16 weeks (Kahleova et al., 2018). Findings from the study showed that body weight, total fat mass, visceral adipose tissue, and insulin resistance were significantly reduced from baseline after the consumption of the high‐carbohydrate low‐fat diet (Kahleova et al., 2018).

Thus, these findings reinforced current dietary guidelines that suggest that other types of macronutrients should gradually replace dietary fat, especially saturated fat. Using carbohydrates to replace fats appears to be a feasible approach to reduce fat intake and prevent cardiometabolic diseases. In food industries, starch‐based fat replacers have also been extensively used in various food products, such as low‐fat yogurt, low‐fat ice cream, confection fillings, salad dressing, and baked goods. The use of starch‐based fat replacers should not only reduce the overall fat content in food, but fat replacers should also have similar physiochemical properties as fats. The physiochemical functionalities of starch‐based fat replacers in foods are discussed in the following sections.

## 3 TYPES AND FUNCTIONALITIES OF STARCH‐BASED FAT REPLACERS

**Figure 1:**
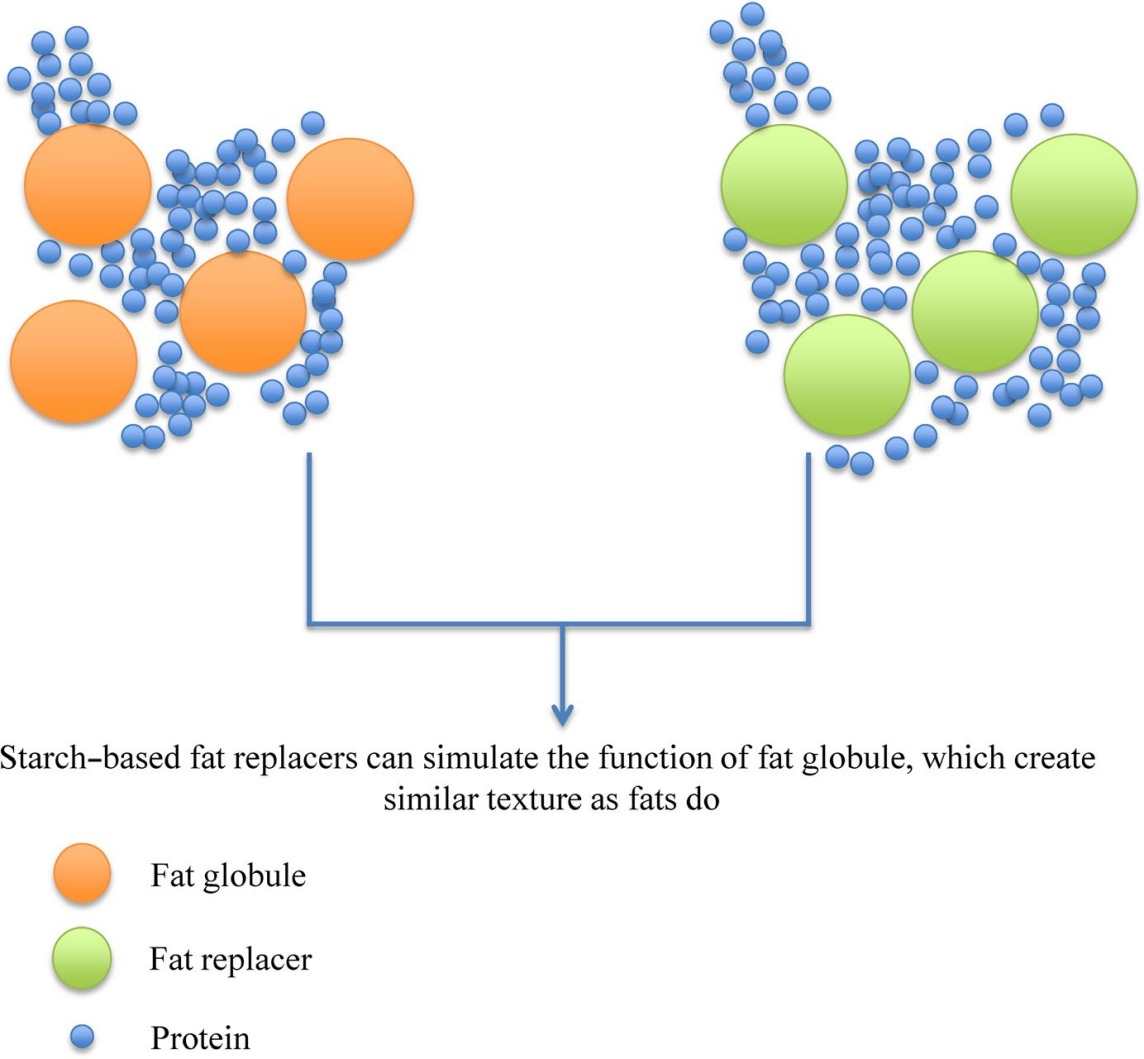
Presumed functionality of starch‐based fat replacers

Fat globules exist in food systems in several forms. For instance, fat globules exist in dispersed or continuous phases that are independent of other components in low‐moisture food systems and therefore contribute to the structural formation of the product (Peng & Yao, 2017). Similarly, fat globules exist as emulsions in high‐moisture food systems and as polymorphic crystalline forms in high‐fat food systems (Peng & Yao, 2017). The unique physiochemical properties of fat in food contribute to various quality‐related properties of food products. For instance, fat significantly affects the viscosity of oil in water emulsions (Peng & Yao, 2017). Viscosity is one of the key parameters associated with the perceived texture of food products. In dilute food systems such as milk, perceived taste and mouthfeel are strongly dependent on fat–protein emulsions. Thus, removing fat globules from the system usually results in a thin texture (Peng & Yao, 2017). On the other hand, fat may also incorporate into protein–starch–fat networks, which provides a tender and soft texture to semisolid/solid food systems such as bread (Peng & Yao, 2017). However, in high‐fat food systems such as chocolate and margarine, fat significantly contributes to the melting property of the food. Therefore, when considering the production of low‐fat food products, simply removing fat from the food may lead to a significant loss of quality of the product. Thus, fat replacers are the ideal alternatives. Figure 1 illustrates the presumed functionality of starch‐based fat replacers in food systems.

Native or modified starch can be considered effective fat replacers because some starch globules have similar sizes and shapes as fat globules. More importantly, native or modified starch has unique physiochemical properties, including thickening, viscous flow, bulking, water holding, and gel formation (Chavan, Khedkar, & Bhatt, 2016). Native starch globules can be dispersed into food systems and generate similar sensory characteristics as fat globules do (Peng & Yao, 2017). However, modified starch is more widely used in the food industry because native starch may not be suitable for all processing conditions, such as low pH, high temperature, and freezing. Cross‐linked starch is a type of chemically modified starch produced by introducing mono‐ or difunctional chemical blocking groups to the starch polymer backbone. Cross‐linked starch does not only exhibit water holding, gel formation, and creating viscous flow, but also can further lower the calorific value of the food as cross‐linked starch has lower digestibility compared to native starch (Peng & Yao, 2017). In addition, substituted starch, such as sodium octenyl succinate, can also have emulsifying properties and be able to carry hydrophobic flavor and nutrients that further improve the quality of the product (Peng & Yao, 2017).

In addition to chemically modified starch, enzyme‐ or acid‐hydrolyzed starch has been invented and used in food systems as fat replacers. Maltodextrins are produced by enzymatic or chemical hydrolysis of native starch. Low‐dextrose‐equivalence (DE < 20) maltodextrins are ideal fat replacers (Chavan et al., 2016). Maltodextrins can be produced from various raw materials, such as cornstarch, wheat starch, tapioca starch, and rice starch (Chavan et al., 2016). However, the functionality of maltodextrins as fat replacers is slightly different than that of native or modified starch because maltodextrins are hydrolyzed products and therefore do not have globular structure. However, the strong water holding capacity of maltodextrins gives it the ability to form gels with water in food systems. Maltodextrin gel has a similar size (1–3 µm) as fat globules, making it an ideal fat replacer and providing fat‐like texture to foods (Peng & Yao, 2017). Interestingly, the maltodextrin gel only contains 1 part maltodextrin and 3 parts water molecules, which can result in a greater energy reduction of the food products (9 cal/g → 1 cal/g). Low DE maltodextrins are believed to be potential fat replacers and have the ability to improve physiochemical properties of food products such as viscosity, lubricity, and softness (Grzelak, Grupinska, Pelczynska, Sperling, & Czyzewska, 2016). There are several commercial maltodextrin‐based fat replacers available on the market, such as Paselli SA2 from potato starch, Maltrin M040 from cornstarch, N‐oil from tapioca starch, and Oatrim from oat starch (Chavan et al., 2016). The USDA has also approved Oatrim‐5, which is a combination of oat beta‐glucan and oat maltodextrins, and the unique combination could deliver additional health benefits to the human body, such as cholesterol‐lowering effects. However, disadvantages associated with using maltodextrin‐based fat replacers should also be noted, including low freeze–thaw stability and low acid and heat stability (Chavan et al., 2016). Table 1 lists some existing commercial starch‐based fat replacers.

**Table 1:** Examples of existing starch‐based fat replacers

Type of starch‐based fat replacer	Producer	Application	Characteristic
Maltrin M040	Grain Processing	Margarine, frozen pastries, salad dressings	Hydrolyzed from corn starch, DE = 5
Paselli SA−2	Avebe	Acidic food	Enzyme‐modified potato starch, DE＜3
*N*‐Oil	National Starch ＆ Chemical	Salad dressings, pudding, artificial sour cream, frozen food	Acidification hydrolysis from tapioca starch
Oatrim	USDA laboratory in Peoria	Mayonnaise, bakery products, cereal, meat products, skim milk powder	Good heat stability, Gel properties, lower serum cholesterol levels
Z‐Trim	USDA laboratory in Peoria	Bakery products, cheese, meat emulsion products	Digestibility, Zero‐calorie, tasteless
Tapioca starch		Low‐fat yogurt	DE 2.0–2.5, taste creamy, translucent
Wheat bran starch		Ice cream	DE 6–10
Potato starch		Ice cream, margarine	DE = 2.5

## 4 APPLICATIONS OF STARCH‐BASED FAT REPLACERS IN VARIOUS FOODS

The first commercial maltodextrin‐based fat replacer called N‐Oil was invented by the National Starch & Corporation in 1984, and later, a modified starch‐based fat replacer was developed by American Maize Products Company in the late 1980s (Jones, 1996). Using starch‐based fat replacers in foods to reduce overall fat and calorie contents has been successfully implemented in various food products. For instance, mayonnaise is a commonly consumed food; however, it contains a substantial amount of fat. Puligundla et al. (2015) replaced 20% of the fat in mayonnaise with either native or cross‐linked rice starch gels. The results showed that reduced‐fat mayonnaises with both types of fat replacers yielded superior sensory quality than the full‐fat control (Puligundla, Cho, & Lee, 2015). In addition, the use of native and cross‐linked rice starch also improved the freeze stability of mayonnaise (Puligundla et al., 2015). Similarly, Parimalavalli and colleagues (2018) investigated the effect of using citric acid‐treated sweet potato starch as a fat replacer on the overall quality of reduced‐fat ice cream. Although the use of modified starch fat replacers compromised the freezing ability of ice creams, reduced‐fat ice cream with 1% fat replacer led to the same sensory acceptance as full‐fat control ice cream and did not affect the microbiological stability (Babu et al., 2018).

In addition, starch‐based fat replacers have been largely implicated in cheese and yogurt processing. In 1993, Mcmahoh and colleagues compared the effects of different types of fat replacers, including Stella made from modified corn starch and protein‐based fat replacers, on low‐fat Mozzarella properties (McMahon, Alleyne, Fife, & Oberg, 1993). The results showed that particles of Stella were evenly distributed between protein matrixes, which improved the melting property and sensory characteristics of low‐fat cheese (McMahon et al., 1993). Extensive research on the use of starch‐based fat replacers in the dairy industry is still ongoing. For instance, Lobato‐Calleros, Ramirez‐Santiago, Vernon‐Carter, and Alvarez‐Ramirez (2014) recently reported that using native or chemically modified tapioca and maize starch has the potential to improve the stability of milk gelled systems in acidified yogurt, improving the overall quality of reduced‐fat yogurt. Octenylsuccinated waxy maize starch‐based fat replacer has also been demonstrated to increase the moisture and water holding capacity of reduced‐fat cheese, improving overall quality (Diamantino, Beraldo, Sunakozawa, & Penna, 2014). Similarly, Sipahioglu and colleagues reported that in combination with lecithin, a commercial fat replacer (N‐lite) made from tapioca starch improved the flavor, texture, and acceptability of reduced‐ and low‐fat feta cheese (Sipahioglu, Alvarez, & Solano‐Lopez, 1999).

However, it should be noted that the abovementioned food products are classified either as concentrated or as semisolid food systems. In food systems, fat globules act as barriers between protein particles and prevent cross‐link interactions between proteins, producing a softer and tenderer texture (Peng & Yao, 2017). However, in solid or dry food systems, fat globules do not usually exist in emulsions. When fats are removed from solid food systems, do starch‐based fat replacers improve the overall quality of the food products, and if so, to what extent? To delineate the efficacy of using starch‐based fat replacers in solid food systems, Balic and colleagues conducted a study in 2016 in which 2% and 4% octenyl succinic anhydride (OSA) wheat and tapioca starch were used as fat replacers in bread formulation (Balic, Miljkovic, Ozsisli, & Simsek, 2016). Findings from the study revealed that up to 4% OSA from either wheat or tapioca starch has the potential to be used as a fat replacer in shortenings in a manner that improves the pasting properties, gel firmness, stickiness, and mixing properties of the dough toward yielding a superior quality of the bread (Balic et al., 2016). Similar to bread, muffins are also an example of a commonly consumed solid‐system‐type food product. Rodriguez‐Sandoval et al. (2017) tested the efficacy of cross‐linked cassava starch as a fat replacer in muffin making. Not surprisingly, up to 8% fat can be replaced by cross‐linked cassava starch without compromising the overall quality or stability of the muffin, yielding a texture similar to that of the full‐fat control (RodriguezSandoval and PrascaSierra. & Hernandez, V., 2017). These findings revealed the efficacy of native or modified starch as fat replacers that can be used in various food products. From Amalean I&II invented by American Maize Products Co to N‐oil invented by A.E. Staley Manufacturing Co, several commercial starch‐based fat replacers are available on the market and play crucial roles in food processing today.

Maltodextrins are starch derivatives that are hydrolyzed from starch and lose the physical properties of starch granules. However, maltodextrin has a strong water holding capacity and is able to form thermoreversible gels with water, creating a similar mouthfeel as fats do. Since the functionalities of maltodextrins as fat replacers are slightly different than those of native or modified starch granules, which require water, solid food systems such as breads and muffins are not ideal applications. Nevertheless, maltodextrins can be used in several food products as fat replacers and can reduce the overall energy content of the products. For instance, confectionery filling contains a substantial amount of dietary fat and is usually considered a high‐fat food. Hadnadev et al. (2014) reported that when 5 g/100 g hydrogenated vegetable fat was replaced with either potato or waxy maize starch‐derived maltodextrins in confectionery fillings, both trained and untrained sensory panelists showed the highest acceptance. The authors also concluded that up to 15% fat could be replaced by maltodextrins without compromising sensory acceptability (Hadnadev et al., 2014). In addition, maltodextrins have been used as fat replacers in ice‐cream making. For instance, Fuangpaiboon and Kijroongrojana (2017) compared the efficacy of various commercial protein‐based and carbohydrate‐based fat replacers in substituting fat from ice cream. Commercial maltodextrins (PD 10) from Nutrition Sc Co. (Thailand) were used as one of the fat replacers in ice‐cream making. The findings suggested that replacing 50% milk fat with maltodextrin did not change any of the sensory attributes compared to those of 8% full‐fat control ice cream; however, higher mouth‐coating and enhanced flavor were observed (Fuangpaiboon & Kijroongrojana, 2017). In addition to the implications of maltodextrins in confectionery filling and ice creams, it has also been demonstrated that maltodextrins are capable of improving the viscosity of low‐fat yogurts (Costa, Frasao, Rodrigues, Silva, and Conte‐Junior 2016). Additional benefits of maltodextrins have also been discovered, such as inhibiting the release of undesirable volatile odors; therefore, maltodextrins can also be used in low‐fat meat processing (Hofman, Van Buul, & Bround, 2016). In addition to reducing the overall energy density of food products by using maltodextrins as fat replacers, physiological benefits such as promoting oral health, reducing appetite, and enhancing brain function have also been linked to the consumption of maltodextrins (Costa et al., 2016). Additionally, a randomized controlled clinical trial reported that daily intake of 10 g maltodextrin for a period of 2 months did not change fasting plasma glucose, fasting insulin levels, or HOMA‐insulin resistance in 25 subjects with type 2 diabetes (Gargari, Dehghan, Aliasgharzadeh, & Jafar‐abadi, 2013).

## 5 CURRENT AND FUTURE MARKET OF STARCH‐BASED FAT REPLACERS

Several authorities, such as the AHA and Health Canada, recommend limiting overall fat and energy intake, and a study conducted in the UK reported that fat intake has decreased significantly over the past 30 years (Pot et al., 2014). Food preferences have been directed toward choosing healthier, low‐energy, and low‐fat foods as heart disease and obesity are significant concerns today. Fat‐based, protein‐based, and carbohydrate‐based fat replacers have been extensively used in the food industry.

According to a recent press release article, the market for fat replacers is anticipated to reach an annual growth rate of 6.5% by 2023, whereas the growth rate may reach 7.2% in societies in Asia–Pacific (Research and, 2018). Similarly, another market report from London stated that the market size for fat replacers is expected to reach $2.79 billion by 2025 (Pharma, 2017). Carbohydrate‐based fat replacers, however, will continue to be the leading product segment with an annual market growth rate of 6.2% from 2016 to 2025 (Pharma, 2017). The surprising market size and growth rate of carbohydrate‐based and other types of fat replacers reflect an excellent means of producing low‐fat and low‐energy food products by using starch‐based fat replacers. In addition, the existing healthier food trend may also lead to a significantly lower rate of mortality and morbidity of CVD and other types of cardiometabolic diseases in near the future, significantly reducing health care costs.

## 6 CONCLUSION

Reducing total fat and energy intake by consuming low‐fat low‐calorie foods has emerged as one of the strategies to prevent CVD and obesity. While several health organizations have recommended that dietary fat intake be strictly controlled, food industries have also been extensively developing and utilizing fat replacers in low‐fat foods. Starch‐based fat replacers, including native starch, modified starch, and maltodextrins, play crucial roles in the low‐fat food industry. These fat replacers mimic the physiochemical and sensory properties as fat globules. Starch‐based fat replacers have been widely used in various food products, such as dairy products, baked goods, salad dressings, and mayonnaise. Through decades of research, starch‐based fat replacers have exhibited promising efficacy in substituting fats without safety issues and are generally considered safe. The market size of fat replacers is also continually growing. However, there is very limited evidence of how starch‐based fat replacers can reduce the risk of CVD and obesity compared to fat in clinical studies. The lack of human intervention trials leads to a poor understanding of other possible side effects that starch‐based fat replacers have while reducing calorie and fat intake in humans, and if there are other side effects, the extent of these effects remains unknown. Therefore, future research should also focus on the physiological efficacy and safety of fat replacers based on human intervention studies while continuing to explore novel fat replacers.

## CONFLICT OF INTEREST

All authors declare no conflict of interest.

## ETHICAL STATEMENT

The present work does not involve any animal and human subjects.
